# Incidence of ocular hypertension after intravitreal injection of anti-VEGF agents in the treatment of neovascular AMD

**DOI:** 10.22336/rjo.2017.38

**Published:** 2017

**Authors:** Andreea Moraru, Gabriela Pînzaru, Anca Moţoc, Dănuţ Costin, Daniel Brănişteanu

**Affiliations:** * “Grigore T. Popa” University of Medicine and Pharmacy, Iaşi, Romania; ** “Prof. N. Oblu” Hospital, Iaşi, Romania; *** Retina Center Iaşi, Romania

**Keywords:** intraocular pressure, intravitreal injection, neovascular AMD, anti-VEGF

## Abstract

**Purpose.** The assessment of the incidence of ocular hypertension over a period of 1 year in patients treated with multiple intravitreal injections of anti-VEGF agents for neovascular AMD.

**Methods.** The study comprised 58 eyes diagnosed with neovascular age-related macular degeneration and receiving PRN intravitreal treatment with anti-VEGF agents (bevacizumab or aflibercept). The follow-up period was 1 year. Intraocular pressure was measured by using the Goldmann applanation tonometry before the intravitreal injection, at 24 hours after the administration of the anti-VEGF agent and at 1 and 4 weeks. Patients diagnosed with glaucoma or who underwent ophthalmic surgery were excluded.

**Results.** The patients received an average of 7.54 intravitreal injections. The mean baseline intraocular pressure was 15.3 mm Hg; 19.8 mm Hg at 24 hours; 17,4 mmHg at 1 week and 14.8 mmHg at 4 weeks after the administration of the anti-VEGF agent. 4 patients required long-term topical hypotensive treatment. Raised intraocular pressure was related to increased frequency of treatment. At 1 year follow up, an average difference of 2.1 mmHg compared to baseline was registered in the cases that have received more than 6 intravitreal injections. By comparison, in the cases treated with a reduced number of doses of intravitreal anti VEGF agent, the difference from baseline was 0,9 mmHg. There were no significant differences in mean IOP depending on the anti VEGF (bevacizumab or aflibercept) agent used.

**Conclusions.** Intravitreal treatment with anti VEGF agents produces a transient increase in intraocular pressure, predominantly immediately following administration, without causing long-term increased values.

Intravitreal anti-vascular endothelial growth factor (VEGF) agents usage is widely spread as primary treatment of many vitreoretinal diseases such as neovascular age-related macular degeneration, diabetic macular edema, macular edema secondary to retinal vein occlusion and other macular edemas [**1**]. The introduction of additional fluid into the vitreous cavity by intravitreal therapy could cause an immediate rise in intraocular pressure (IOP). This transient, short-term IOP elevation (lasting up to 30 minutes) after intravitreal anti-VEGF therapy has been studied by many authors [**2**].

Bevacizumab (Avastin), a monoclonal antibody targeting vascular endothelial growth factor (VEGF), was approved by the Food and Drug Administration (FDA) for systemic administration in patients with metastatic colon cancer. Bevacizumab was first injected intravenously to treat age-related macular degeneration (AMD). The use of intravitreal bevacizumab in treating ocular diseases was first reported in 2005 for choroidal neovascularization (CNV) caused by AMD. One of the side effects is related to the rise of intraocular pressure (IOP), which can be transient or permanent [**3**]. 

Aflibercept (Eylea) is a fully human fusion protein consisting of portions of VEGF receptors 1 and 2, which binds all forms of VEGF-A, along with the related placental growth factor, which the drug blocks. FDA approved aflibercept for the treatment of neovascular (“wet”) age-related macular degeneration (AMD) in 2011 [**4**].

In all cases, an effective therapy with anti-VEGF antibodies can only be achieved by repeated intravitreal anti-VEGF injections. In general practice, three intravitreal injections of anti-VEGF antibodies are given at every 4 weeks in the initial upload phase, followed by further intravitreal injections of anti-VEGF antibodies if the lesion persists or increases. Therefore, a monthly ophthalmologic checkup is needed to ensure a correct treatment interval with anti-VEGF antibodies [**5**]. The recommended dose for Aflibercept administered by intravitreal injection is once at every 4 weeks for the first 3 months, followed by intravitreal injection once at every 8 weeks [**6**].

## Material and methods

58 eyes diagnosed with neovascular age-related macular degeneration and receiving „Pro Re Nata” (PRN) intravitreal treatment with anti-VEGF agents (bevacizumab or aflibercept) were included in this study. The follow-up period was 1 year. 

Inclusion criteria consisted in age between 65 and 85 years, initial IOP < 21 mmHg, ability to understand and sign the consent form, and ability to follow the scheduled visit protocol. The exclusion criteria consisted in: open-angle or angle-closure glaucoma, suspected glaucoma (IOP > 21 mmhg and/ or cup to disc ratio > 0.5), currently receiving a systemic beta blocker, previously receiving intravitreal injection of any medication (steroid, ganciclovir, and anti-VEGF agent), current use of steroid eye drops, and any ocular surface disease precluding a reliable IOP measurement.

The follow-up protocol of patients with exudative AMD treated with anti-VEGF required strict monthly follow-up visits with complete ocular examinations, including IOP measurements.

Intraocular pressure was measured by using the Goldmann applanation tonometry before the intravitreal injection, at 24 hours after the administration of anti-VEGF agent at 1 and 4 weeks. Ocular hypertension was defined as intraocular pressure over 21 mmHg. Patients diagnosed with glaucoma or who underwent ophthalmic surgery were excluded. All the patients received a loading dose, which consisted of one monthly-administered injection, for three months.

The patients were divided into two groups according to the injection type:

• 50 (86%) patients received bevacizumab 

- 36 (72%) patients received the injection at an interval of less than 8 weeks (range 4-7 weeks) after the initial loading dose

- 14 (28%) patients received the injection at an interval of more than 8 weeks (range 8-12 weeks) after the initial loading dose

• 8 (14%) patients received aflibercept (once at every 2 months after the initial loading dose).

## Results

58 eyes of 58 patients diagnosed with neovascular age related macular degeneration were analyzed in the present study. The mean age was 69 years (ranging from 65–85 years of age) and most of the patients were women. The study included 36 phakic eyes (62%) and 22 pseudophakic eyes (38%).

**Fig. 1:**
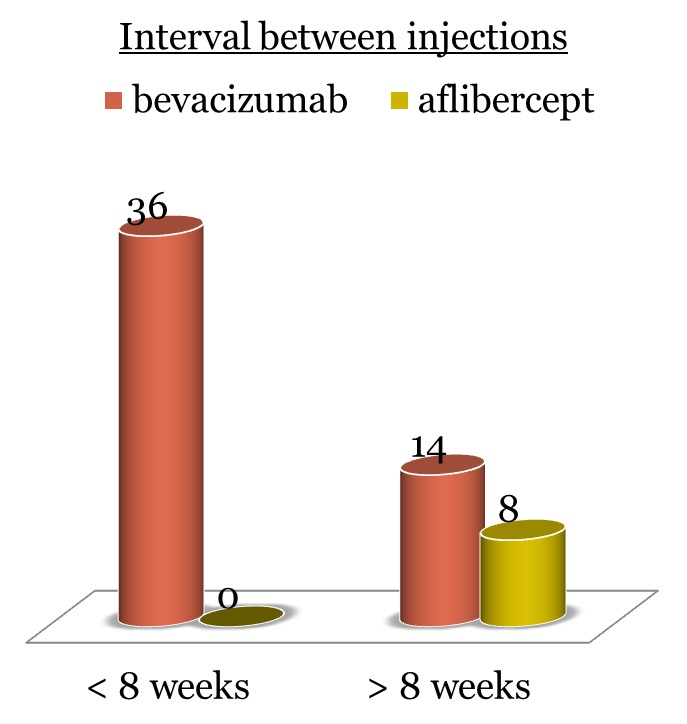
Frequency of treatment after the administration of the initial loading dose of bevacizumab/ aflibercept

Most patients received intravitreal bevacizumab injection (86%) and the rest were treated with intravitreal aflibercept (14%). All the patients were treated with the standard dose of Avastin or Eylea administered intravitreally. Most patients, 72%, received the bevacizumab injection at an interval of less than 8 weeks after the initial loading dose. The patients receiving aflibercept were treated according to the recommended protocol (once at every two months after the initial loading dose) (**Fig. 1**).

The patients received an average of 7.54 intravitreal injections during the study period, with limits between 3 and 10 injections. The only complications seen post injection were discreet corneal edema, conjunctival congestion, subconjunctival hemorrhage.

IOP was monitored for all patients prior to treatment and at all follow-up visits. Sustained IOP elevation was defined as an IOP of 21 mmHg or greater, combined with a rise of 6 mmHg or more lasting for 30 days or more, compared to baseline. The average IOP rose from 15.3 mmHg (range 8-15 mmHg) before the initiation of injections to 19.8 mmHg (range 16-28 mmHg) after the treatment.

**Fig. 2:**
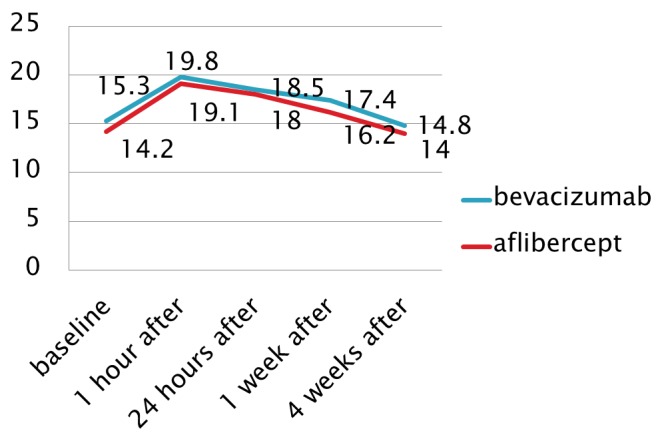
Variation of mean IOP values after the intravitreal injection

No significant differences were found in IOP elevations between the two groups depending on the anti VEGF agent used (bevacizumab or aflibercept) (**Fig. 2**), although bevacizumab seemed to induce a slightly higher IOP level than aflibercept. 

The raised intraocular pressure was related to an increased frequency of treatment. Increased levels of the IOP were observed in patients who received more than six injections and those in whom the frequency of the treatment was of less than eight weeks. The interval between injections was the most remarkable risk factor for the IOP elevation identified in this study. The IOP elevation was greater when the average interval between the injections was of less than eight weeks compared to the average intervals of eight weeks or more.

**Fig. 3:**
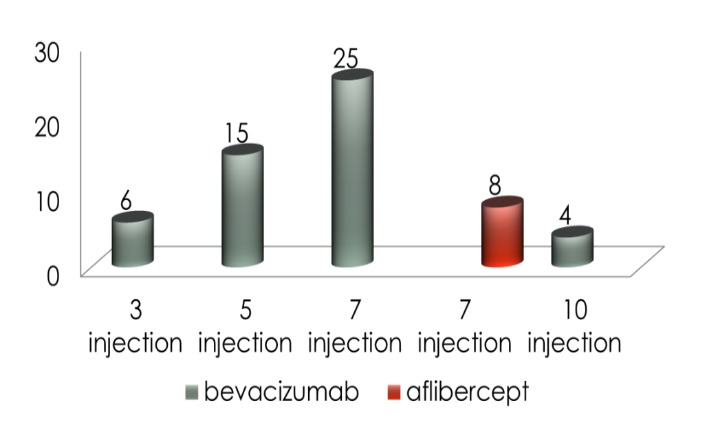
Number of patients in relation with the number of intravitreal injections of bevacizumab/ aflibercept received

At 1 year follow up, an average difference of 2.1 mmHg compared to baseline was registered in the cases that have received more than 6 intravitreal injections. By comparison, in the cases treated with a reduced number of doses of intravitreal anti VEGF agent, the difference from baseline was 0,9 mmHg.

None of the patients had to undergo glaucoma surgery or discontinue the participation in the study following uncontrolled IOP. Four patients needed topical hypotensive treatment during the follow up period due to the maintenance of IOP at levels higher than 25mmHg.

## Discussions

The intravitreal injection of anti-angiogenic agents has assumed an important leading role in the treatment of a growing number of ophthalmologic conditions since 2005, when the intravitreal use of bevacizumab was first reported [**7**]. 

Evidence collected in recent years has demonstrated that intravitreal injections may produce short- and/ or long-term elevations in IOP. The possible mechanisms for a sustained IOP elevation after the intravitreal injection of anti-VEGF agents are not very well understood. Anti-VEGF agents may directly damage the trabecular meshwork, so one possible mechanism of IOP elevation is inflammation, such as drug-induced trabeculitis or uveitis [**8**].

Another mechanism of anti-VEGF-induced IOP elevation is considered an underlying inflammatory mechanism or an immunological reaction that damages the aqueous humor outflow pathways. Besides that, a traumatic mechanism after frequent injections may lead to a disruption of the anterior hyaloid or zonules and allow access for high molecular weight proteins to enter the anterior chamber, resulting in increased IOP [**9**].

The IOP spike that occurs after the intravitreal injection of anti-VEGF agents is usually transient, with IOP returning to a safer range (< 25–30 mmHg) within 1 hour, without topical hypotensive medications, in most patients [**10**].

A prolonged elevation was found in 4 out of 58 patients treated with recurrent intravitreal Eylea or Avastin who needed topical hypotensive treatment during the follow up period. None of the patients who suffered from IOP elevations had a history of glaucoma or ocular hypertension. 

Some authors reported an increased risk of IOP elevation in patients receiving >29 injections compared to patients with <12 injections [**11**]. Moreover, an increased risk of IOP elevation was also found in the groups with a higher number of injections.

Initial IOP elevation varied from 1 hour, 1 week, and 3 months after treatment. In 3 eyes, the IOP returned to baseline levels after 1 month, even when receiving monthly injections. A possible explanation for the low rate of sustained IOP elevation could be that 64% of the patients in our study did not receive monthly injections, but rather, received an extended treatment regimen. It is possible that the increase of the interval between injections will allow the anti-VEGF medication to be cleared from the eye [**12**].

The type of injected drug (Avastin versus Eylea) did not have a statistically remarkable influence on the difference in IOP, but a slightly higher IOP was found in eyes receiving bevacizumab. The limitations of this study include its retrospective nature and a short follow-up period, which implied a limited number of injections. 

## Conclusion

In conclusion, most eyes in our study achieved a normalization of IOP within 24 hours without the need for any immediate intervention. 

A close monitoring of IOP is required in patients undergoing intravitreal Avastin injections, especially in those patients receiving frequent injections, to prevent further ocular damage, in addition to the underlying retinal disease. A prompt diagnosis and treatment can prevent the potential loss of vision.
